# A Review of Methodologies for the Rapid Analysis and Quantification of Gases in Solid Media

**DOI:** 10.3390/s24237777

**Published:** 2024-12-04

**Authors:** Chu Zhang, Leizi Jiao, Yibo Wei, Feng Bao, Rui Guo, Daming Dong

**Affiliations:** 1 School of Optoelectronic Engineering, Guilin University of Electronic Technology, Guilin 541004, China; 13241263683@163.com; 2 Research Center of Intelligent Equipment for Agriculture, Beijing Academy of Agriculture and Forestry Sciences, Beijing 100097, China; jiaolz@nercita.org.cn (L.J.); baof@nercita.org.cn (F.B.); guor@nercita.org.cn (R.G.); 3 Research Center of Information Technology, Beijing Academy of Agriculture and Forestry Sciences, Beijing 100097, China; weiyb@nercita.org.cn

**Keywords:** gas detection, porous media, extraction measurement, in-medium scattering spectroscopy, gas sensing probes

## Abstract

Gas sensors are essential measurement devices that have found extensive applications across various fields, including industry, agriculture, ecological and environmental monitoring, military operations, and biomedical research. Numerous sensing methods based on a diverse range of principles—including optics, electrochemistry, and semiconductors—have been used in the development and manufacture of gas sensing technologies. However, the measurement of certain gases remains challenging when using current sensing techniques and sensors; this is particularly true for the gases that are present in solid media. For example, the nitrous oxide that is emitted from soil is often trapped within soil pores, while a significant portion of the ethylene that is released from fruit dissolves within the flesh of the fruit itself. Measurement of the gases in these situations poses difficulties when using conventional gas sensing methodologies. To enable the detection of these elusive gases, scientists and engineers have devised a variety of specialized approaches over the past two decades. In this review article, we summarize several of these sensing methods—including extraction measurement techniques, in-medium scattering spectroscopy, and the use of micro-nano gas sensing probes—and discuss their respective advantages and disadvantages, along with emerging trends in the development of these techniques.

## 1. Introduction

In the initial stages of gas measurement and sensing technology development, the primary application of the technology was monitoring toxic gases in the environment, thus allowing timely hazard warnings to be provided. However, as our understanding of gases has evolved, it has become increasingly evident that appropriate gas analysis can yield a wealth of valuable information. A more nuanced comprehension and detection of the components of gases can provide a deeper insight into environmental conditions. With ongoing advancements in gas sensing technology, there is escalating demand for enhanced sensitivity and accuracy in gas monitoring systems that can adapt to a diverse range of complex environmental scenarios. Consequently, gas sensing technology now plays a pivotal role in various sectors. In the industrial domain, it is used to monitor harmful gases used during production processes to safeguard operational safety and to oversee manufacturing procedures to maintain the required product quality. In agriculture, gathering environmental gas data that are pertinent to crop growth aids in making timely adjustments in the field and implementing relevant measures that will ultimately enhance crop yields. In the biomedical field, the unique odors that are associated with diseases correspond to specific disease types, meaning that gas sensors can be used to support diagnoses. In contemporary society, gas sensors, as important devices that convert gaseous information—e.g., gas concentration and type—into recognizable formats for instruments or computers, play important roles in daily life and production activities.

**Figure 1:**
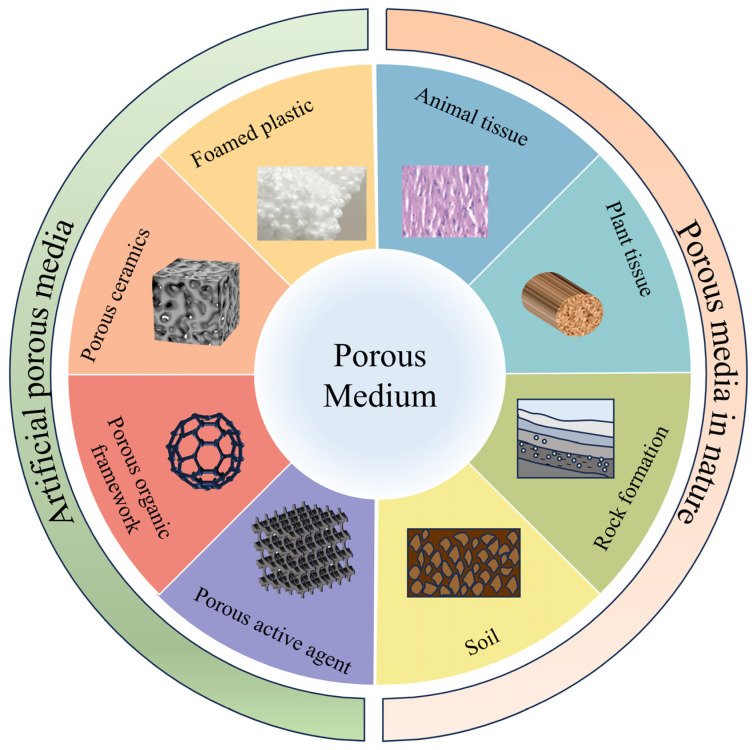
Common porous gas-bearing media.

However, in complex environments in which gases are present and are distributed, there is a scenario in which the gases permeate solid media and then occupy the material’s pores. Investigation of the behavior of the gases within these pores is essential to understand the characteristics of gas-containing media and the conditions required for monitoring the gases within them. Various types of porous media that contain gases can be found in nature, as illustrated in Figure 1. Natural porous media include soil and biological tissues, where gases in the soil can be hidden within soil pores, and where the gases generated by biological activities fill spaces within the tissue. Artificial porous media, such as porous alumina materials, foams, and porous catalysts, often have their pores filled with gases to meet industrial processing and packaging environment requirements. Therefore, gas detection does not only reflect the characteristics of the gas-containing media, but can also provide information about their environment, thereby expanding the scope of analysis. This capability frequently intersects with materials science applications, in which porous materials are used for gas sensing purposes [1]. Nevertheless, because of the diverse and potentially irregular pore structures that exist in solid media containing gases, the challenges related to gas sampling and measurement can be summarized as follows. First, it is difficult to collect gas from porous media; methods based on squeezing the sample to release the gas or using a probe to invade the sample are destructive to the sample, and may also affect the sample itself and the gas in it. The collection method itself can also easily affect the composition of the gases within a sample. The gas content within the pores is minimal, which imposes requirements on the detection limit of the measuring instrument. Furthermore, if the analysis and measurement time takes too long, then the gas components within the pores may change, and thus it is often difficult to adapt conventional sampling and measurement methods to these special gas-bearing media. Therefore, to address this situation, it is particularly important to select appropriately rapid on-site measurement methods to ensure that timely and accurate measurements are taken.

This article summarizes the different instrument types used to perform field gas analysis and the countermeasures taken by gas sensors during the analysis of gases in solid media. The applications of postharvest detection, invasive detection, and nondestructive in situ detection methods are analyzed separately. First, concerning analysis methods that involve sampling before detection, the analysis in this work includes gas chromatography mass spectrometry (GC-MS), which is a reliable method for gas quantitative detection, along with mass spectrometry methods such as proton transfer reaction mass spectrometry (PTR-MS) and ion mobility spectrometry (IMS). These methods offer significant advantages for the detection of gas environments with more complex components. Sensor-based measurement methods based on electrochemical sensors and other sensor types can play important roles in measuring specific gas types in different media. Second, in terms of invasive measurement methods, improvements in and the further development of probes have provided convenient and rapid on-site measurement possibilities. Micro-nano probes such as fluorescent probes and invasive biosensors are of major significance for the real-time monitoring of biological activities. Finally, among the many measurement methods based on absorption spectroscopy techniques, non-invasive scattering medium gas absorption spectroscopy (GASMAS) technology offers important advantages for the detection of gas-bearing porous media, and its principles and methods are analyzed in detail here. Regarding the methods used to detect gases in solid pores, this article classifies different types of gas detection technologies based on their sampling and detection methods, and the sampling methods and corresponding application scenarios after sampling are analyzed and summarized. Finally, the measurement methods for gas in solid media are summarized and their prospects are discussed.

## 2. Measurement Methods After Field Sampling

### 2.1. Instrumental Analysis Methods After Sampling

**Figure 2:**
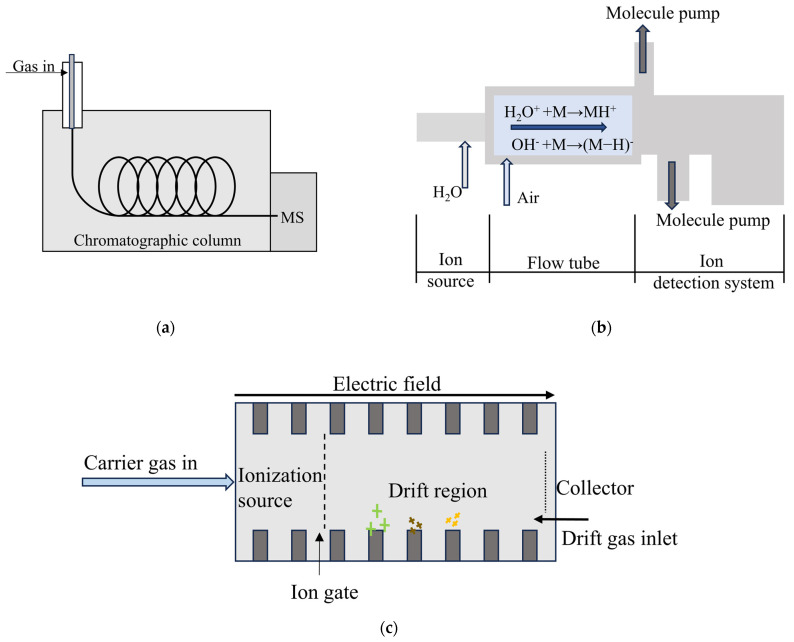
(**a**) Schematic diagram of GC-MS; (**b**) schematic diagram of PTR-MS; (**c**) schematic diagram of IMS.

Laboratory analysis methods such as GC-MS have long been the standard methods for both the qualitative and quantitative detection of gases, and the sensitivity and accuracy of these methods have been recognized for use in long-term applications. Similarly, when addressing the analysis of gases in solid voids, the most straightforward approach involves the sampling and subsequent analysis of the gases via standard methods such as GC-MS. In GC-MS, the GC part is responsible for the separation of gas samples in a chromatographic column, and its high separation ability is conducive to the further identification and analysis of samples by mass spectrometry. Its schematic diagram is shown in Figure 2a.

However, sampling poses challenges for most gases contained within solid matrices because of their low concentrations and the difficulty involved in disrupting the solid voids. Generally, there are two types of sampling methods: active and passive. Passive sampling entails allowing gases to flow automatically into a sampler, which often requires specific conditions. Consider the example of gas measurement in soil crevices: Passive sampling takes a long time, with periods of several days being required to complete the collection process [2], whereas active sampling methods usually use a sampling probe connected to an air pump to extract the gas from the soil and transfer it into a gas sample storage device, e.g., an adsorption tube, sampling tank, or air bag, for sealing [3]. With reference to existing standard methods, such as the U.S. Environmental Protection Agency (EPA) method TO-15, the soil gas samples obtained are collected, stored, and transported to the laboratory for analysis. It is important to note that although passive sampling takes longer, it does not disturb the natural environment of the gases. Active sampling, however, may alter the gas release patterns, leading to discrepancies when compared with their natural state; this is particularly important with respect to the volatile gases released from soil voids.

In practical scenarios, one common constraint is that the gas collected often changes during storage. This alteration, which is due to factors such as adsorption, causes significant errors when the samples are sent to the laboratory for analysis. To address this issue, researchers have explored numerous solutions, with gas pre-concentration and rapid field mass spectrometry being notable methods. The most commonly used pre-concentration methods are defined by their sampling processes, and the main sampling methods for gases in solids include static headspace (HS), purging–capture (PT), headspace–solid-phase microextraction (SPME), and other headspace technologies. The selection of the pre-concentration method is dependent on the concentration of the volatile compounds to be measured and the substrate type selected. Headspace–SPME is a widely used sample treatment technology. This technique has been used effectively for the quantitative detection and treatment of chlorinated hydrocarbons, chlorobenzene, polycyclic aromatic hydrocarbons, and herbicides in soil [4]. Typically, SPME processes use a fiber coated with a stationary phase, which is placed in the headspace of the sample to achieve the selective adsorption of gas molecules, and then thermal desorption is performed on it during detection [3]. As an alternative method, a needle capture extraction device with a particle extraction medium captures the VOCs from the collected soil samples and passes them directly into the air stream. This device can then be connected directly to a GC detector for thermal desorption, thus enhancing process efficiency [5]. Additionally, in targeted compound extraction, the extraction steps are structured to minimize the time required to perform the extraction process and allow quicker sample detection. Some researchers have introduced a method for the extraction of chlorinated compounds in soil that is known as QuEChERS [6]. This method refines the traditional extraction steps, simplifies the process, and reduces the extraction time required to approximately 10 min. Extraction efficiency is usually related to the composition of the extractant, in addition to being affected by environmental conditions such as temperature and humidity. On the one hand, various gas pre-concentration methods are applied to ensure that the gas samples maintain their integrity during transport and transfer. On the other hand, these methods also enable the instruments to attain lower detection limits, which is particularly evident when using portable GC-MS.

Portable GC-MS enables quantitative sample analysis in field settings. The spectrometer features rapid analysis capabilities and high sensitivity, thus allowing the simultaneous identification of multiple organic compounds [7]. Because of detection limits and sensitivity constraints, the pre-concentrator plays a crucial role in the operation of this portable instrument. This means that the selection of an appropriate pre-concentration method is particularly significant. Unlike traditional laboratory GC-MS, the portable version emphasizes simplicity and speed during the pre-treatment process and prioritizes ease of sampling and the potential for automation. Consequently, the pre-concentrator design focuses on miniaturization in combination with the capability to withstand high-temperature heating and low-temperature desorption. The selection of the adsorbent typically favors materials that provide a robust adsorption capacity. Foam adsorbents demonstrate superior adsorption capacities and thermal transfer efficiencies when compared with conventional particle and film adsorbents, thus enhancing the pre-concentration performance of portable GC-MS [8]. Additionally, the selection of extraction solvents with higher extraction efficiency, coupled with precise temperature control, can contribute significantly to boosting the pre-concentration efficiency. Notably, portable GC-MS has been used to detect trichloroethylene (TCE) in soil samples by using solid-phase microextraction (SPME). The incorporation of a small amount of ethanol and reducing the exposure temperature to approximately 4 °C caused the extraction efficiency of SPME to improve markedly. As a result, the detection limit of the portable GC-MS method decreased from around 1000 µg/m^3^ to approximately 100 µg/m^3^ [9]. In summary, the adsorbent material and extraction solvent choices affect the extraction efficiency significantly. The use of adsorbents with a high adsorption capacity and solvents with superior extraction efficiency constitutes an effective strategy for the enhancement of the detection limits of portable GC-MS instruments.

Regardless of whether a laboratory GC-MS instrument or a portable GC-MS instrument is used, the method used for the sampling of gases in soil is also applicable to some extent to gases within sediments or other porous media. For example, when gaseous mercury in snow is measured in situ, a traditional active sampling method is used to sample the gas contained in the snow gap. A probe is inserted deep into the snow, an air flow is applied for sampling, and the probe is connected to a mercury analyzer, which can analyze the gaseous mercury concentration [10,11]. However, for the more complex volatile organic compounds that may exist in snow cover, the use of GC-MS for component analysis gives more reliable results [12]. This also has application prospects in terms of finding underground leaks in time, dealing with pollution by detecting and preventing gas leakages at their source.

Another type of gaseous medium that is commonly used in research is plant tissue. It is often important to detect volatile organic compounds (VOCs) within plant tissues. This detection process helps to clarify the physiological processes of plants and to assess their stress levels based on changes in the VOCs. These changes can reveal how environmental conditions affect plant health. GC-MS stands out as a widely used technique for the identification of the bio-volatile organic compounds (BVOCs) produced in plant tissues [13]. When using nondestructive sampling methods to measure plant VOCs, GC-MS provides high sensitivity through a thermal desorption approach. First, it is necessary to collect the gas that has been volatilized by the plants of interest. Common nondestructive methods include static headspace sampling, dynamic headspace sampling, and direct contact measurement [14]. In both the static and dynamic headspace sampling methods, the plants are placed in a sealed environment. The VOCs then accumulate in the closed headspace before an analysis is performed. Direct contact measurement involves the use of an adsorption tape to capture and extract any gases that have not yet been volatilized into the atmosphere. This method allows for repeated in situ gas sampling from the same plant, thus enabling simultaneous collection from different parts to be realized without causing harm to the plant. Ultimately, these approaches enable researchers to understand the dynamic responses of plants to stress or damage [15].

In summary, GC-MS has become widely used as an effective method for the detection and analysis of gases in solid media. The development of appropriate sampling methods and pre-extraction techniques for use in different media, along with the application of portable GC-MS instruments, has led to a continuous series of reductions in instrument detection limits and produced more reliable analysis results, thus making GC-MS increasingly suitable for on-site analysis applications.

### 2.2. Online Sensor Measurements After Sampling

For the determination of VOCs, GC-MS is the preferred off-line measurement method. Although it offers obvious advantages for the detection of complex compounds, its limitations are mainly reflected in the pre-concentration technology that must be used for processing and the time required to perform a single analysis being long. The manipulation and interpretation of results require a great deal of knowledge and technical experience, and the accuracy of the results themselves is easily affected by the operating process and environmental conditions. Online measurement techniques can achieve rapid analysis of the target, and the main commonly used online measurement methods are described in the following subsections.

#### 2.2.1. Field Measurement Method Based on Mass Spectrometry and Ion Mobility Spectrometry

Proton transfer reaction mass spectrometry (PTR-MS) allows the rapid online quantification of VOCs without requiring a pre-concentration step. The high sensitivity and low detection limits of this method can reach pptv levels. PTR-MS uses a proton transfer reaction to generate ions, and the mass-to-charge ratio of the ions is analyzed by a mass spectrometer to determine the compound type and concentration in the sample. Its schematic diagram is shown in Figure 2b. This swift detection capacity supports real-time analyses in various fields, thus allowing quick monitoring and post-sampling assessment. For example, when investigating the VOCs emitted by plant roots in soil, researchers generally perform dynamic headspace collection directly over the soil that contains the roots. They then use PTR-MS to conduct real-time VOC analysis, allowing them to track the changes in sulfide emissions that occur when the roots experience stress from herbivores [16]. In addition to its direct role in gas analysis, PTR-MS is often integrated with other methodologies to enhance measurement resolution quality. Researchers have successfully coupled PTR-MS with a time-of-flight (TOF) analyzer to yield PTR-TOF-MS. This configuration achieves improved mass resolution for the detection of complex VOC mixtures, increasing from approximately 1200 m/Δm to over 6000 m/Δm, while also maintaining the benefits of direct sampling and real-time measurement [17]. Additionally, this method achieves lower detection limits (as minimal as a few pptv) and enhanced sensitivity when compared with PTR-MS alone. PTR-TOF-MS has also enabled the detection of microbial gas emissions in soil, thus providing insights into microbial activity based on the measured gas concentrations, which reflect varying soil enzyme activities [18]. As a prevalent method for VOC investigation, PTR-MS can analyze compounds like acetaldehyde, acetone, and acetic acid efficiently to capture dynamic emission changes over time [19]. Nevertheless, it is essential to note that PTR-MS may not be able to identify isomeric compounds effectively. To allow specific isomer detection, running GC-MS alongside PTR-MS is advised [13]. Previous studies have used fast GC systems alongside PTR-TOF-MS to enable the effective separation of isobaric isoprenoid compounds that PTR-TOF-MS cannot analyze directly [20]. In addition, if quantitative analysis is required when using PTR-MS, pure compounds of the measured gas must be provided, which adds some inconvenience to the quantitative detection process.

For real-time gas detection, ion mobility spectroscopy (IMS) is a widely used online method. Ion mobility spectroscopy is an effective gas-phase analysis technique that separates and identifies different substances by measuring the mobility rate of ions in the electric field. A schematic diagram is shown in Figure 2c. When IMS is applied to the analysis of VOCs in soil, researchers can assess the headspace of the soil samples directly. Notably, some researchers have developed subsurface ion mobility spectrometers that use probes that have been installed underground [21]. These probes allow the real-time detection and separation of contaminants such as trichloroethylene and tetrachloroethylene within the soil gases and have achieved detection limits as low as 1–10 ppbv. To enhance the performance of IMS and address its limitations in isomer discrimination applications, researchers frequently integrate IMS with complementary techniques such as gas chromatography and mass spectrometry to yield coupled devices that include GC-IMS and IMS-MS. Various sampling methods and sample pre-treatment devices have been proposed to streamline the sampling and testing processes. Specifically, solid-phase microextraction (SPME) has been combined with IMS (SPME-IMS) to allow the effective headspace sampling of gases from soil, thus aiding the analysis of precursors or degradation products of chemical warfare agents [22]. Alternatively, coupling IMS with a membrane interface probe (MIP) enables effective capture of gas samples while excluding irrelevant liquids, thus enabling ion mobility spectrometer analysis [23]. Field sampling has also advanced with the integration of a needle trap device (NTD), which is paired with GC-IMS to perform real-time measurements, with detection limits of less than 0.7 ng being achieved [24]. When deployed in series with a mass spectrometry technique, IMS-MS accurately distinguishes compounds that share identical *m*/*z* ratios but have differing structures, thus making the technique suitable for the analysis of intricate copolymers [25]. Overall, IMS has demonstrated high sensitivity to various chemical compounds [26]. The method’s real-time measurement capability, coupled with its cost-effectiveness, has led it to become a valuable gas detection and analysis tool, although it may yield significant errors during complex mixture detection. These limitations have been addressed through integration with additional detection technologies that have enhanced its reliability in field applications.

#### 2.2.2. Field Measurement Methods Based on Sensor Arrays and Electrochemical Sensors

**Figure 3:**
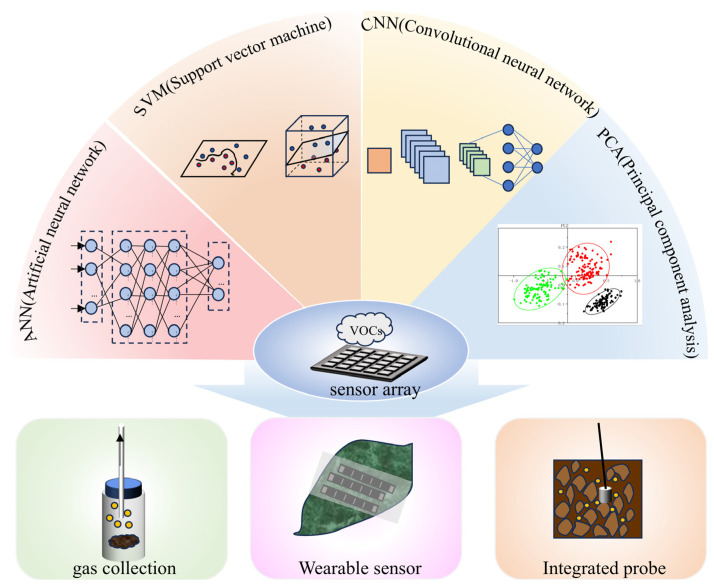
Schematic diagram of a sensor array and several application methods.

In addition to the use of mass spectrometry methods, devices that are also commonly used to monitor gases include, for example, electronic noses based on sensor arrays. Electronic noses can analyze the headspaces of samples such as soil to allow the assessment of VOCs. De Cesare and colleagues used an electronic nose with eight chemical sensors to monitor oxygen and carbon dioxide, and distinguished the different metabolic and growth activities of microorganisms by monitoring the soil headspace concentrations [27]. The advantages of electronic noses lie primarily in their ability to monitor various gas categories simultaneously, rather than simply focus on a single substance. However, their limitations include reduced detection capabilities for both complex gas mixtures and low-concentration gases, in addition to their susceptibility to interference. In practice, sensor arrays are often combined with computer analysis methods such as ANNs (artificial neural networks), SVMs (support vector machines), CNNs (convolutional neural networks), and PCA (principal component analysis), as illustrated in Figure 3, which greatly improve the accuracy of the analysis.

With advancements in materials science, the number of configurations available for sensor arrays has expanded. Traditional rigid sensors have poor flexibility due to their large size and poor applicability to different environments. This gives wearable sensors a unique advantage when it comes to surface attachment measurement. They can directly measure the gas in the pores near the surface of an object. A team led by Zheng Li developed a wearable sensor array made from graphene that was designed to detect VOCs emitted by plants [28]. By attaching this chemical resistance sensor to the leaves of tomato plants, the gas signals generated by the leaves can then be detected and recognized. The array was able to detect various plant volatiles at low ppm concentrations and classified 13 common plant volatiles with an accuracy of more than 97%. This graphene array sensor demonstrates greatly improved detection sensitivity and can detect the physiological activities of plants more accurately than previous techniques. Similarly, gas in plant leaf gaps can be measured using wearable plant sensors. Ref. [29] uses a composite material of conductive polymer microcrystals and platinum nanoparticles; in this way, methanol emissions on plant leaves can be directly monitored under field conditions with detection limits up to sub-ppm. Wearable sensor arrays can also be attached to human skin to detect skin odor in order to analyze and distinguish the sweating of the human body, and thus understand the health status or activity of a person [30,31]. In food research, wearable chemical resistance sensors established using a new flexible nanocomposite material can be used to detect H_2_S produced inside eggs by sticking to the surface of the egg. The detection concentration range is 0.1–1 ppm, so as to realize the dynamic monitoring of the internal deterioration of the egg [32]. In addition, a chemical resistance ethylene gas sensor based on SWCNTs/PdNPs/Cu-MOF-74 nanocomposites can be used to determine the spoilage of kiwifruit and detect the ethylene gas produced by it [33].

For sensor arrays and new wearable sensors, the advantage is that the real-time detection of gas can be realized, but is easy for the monitoring environment to be interfered with by factors other than the detection object, such as temperature, humidity, and other environmental factors. Moreover, the application is highly targeted and the collection of data is singular, so the application is not extensive. In the future, sensors that are more adaptable and can be used in different scenarios will be needed.

In short, for samples such as gases in soil, methods based on rapid measurements after sampling offer a range of different advantages. The advantage of rapid online measurement lies in the selection of an appropriate measurement method based on different measurement objects. Current researchers frequently use the headspace dynamic acquisition technique for the sampling of gases within the media that contain them. To ensure the reliability of the results, many studies validate their measurements using traditional GC-MS, thereby confirming the credibility of these online detection methods. Although portable instruments have been developed to a certain extent to date, because the performance of field instruments is generally weaker than that of laboratory instruments, it is important to pay close attention to the requirements of the sampling methods and the measurement environment when using these measuring techniques [34]. The development of new wearable sensors has expanded the range of objects available for gas monitoring and has important application prospects in biological detection. However, the problems of complex material synthesis and high cost still need to be solved.

## 3. Invasive Sensor Measurement Methods

### 3.1. Gas-Sensitive Electrodes and Probes

In addition to the use of different sampling methods to extract a gas from a solid medium and then detect it, it is also a feasible idea to use an intrusive electrochemical sensor with a gas-sensitive electrode, which is inserted directly into a gap in the measurement object for the detection of gas, or to use a probe to detect the gas. This enables the in situ detection of gases in their respective voids. When performing gas analysis in substrates such as soil, the integration of an electronic nose module into the soil probe enables direct contact with the gases and enables easy measurement. This method can detect the chlorinated hydrocarbons, aromatic hydrocarbons, and fatty hydrocarbons that are present in the soil, achieving detection limits below 1 mg/m^3^ and enabling the differentiation of these pollutants for accurate pollution classification and screening [35]. Soil gas detection probes not only extract the gases from soil voids for transmission to analytical instruments, but also incorporate hydrophobic membranes that enhance gas diffusion—an established technique that improves probe performance [36]. Various methods have been used to improve probes to allow them to complete the sampling process and aid in the rapid realization of detection.

To allow the detection of known specific gases, corresponding gas sensors can also be selected. For detection of gases in biological tissues, most of the commonly used sensors are electrochemical sensors. Electrochemical sensors have low operating powers, high response speeds, and small sizes, which can enable direct, real-time rapid measurements of biological samples and can also be used to detect gases in animal and plant cells. NO plays an important role in biological processes, and thus NO concentration has been measured using electrochemical sensors in studies of animal cell biology. For example, to study the NO concentration in plant and animal cells, electrode elements and devices were used to conduct electrochemical NO detection in a cell suspension of tobacco plants [37], but this method required the destruction of the plants, and in vitro measurements are also prone to environmental interference. The electrodes of electrochemical sensors can be inserted into cells using a computer-controlled micromanipulator for the direct measurement of NO concentration in both plant and animal cells. In the case of fruit, NO levels can be measured by simply inserting the electrode directly into the fruit. Electrochemical sensing has also proved to be highly effective in the analysis of the growth status of plants. Arasimowicz et al. observed wound sites on geranium leaves using NO-selective microelectrodes. They recorded the occurrence of a notable surge in NO concentration within 5 min after the injury was sustained, indicating that NO plays an essential role in the recovery of the physiological state of damaged tissue [38]. Existing electrochemical sensors are extremely small and can detect NO concentrations without causing significant damage to the surrounding tissues in the body; they have been used previously to detect NO concentrations in rat brains. In addition to the electrochemical sensor for NO, an electrochemical sensor with a platinum electrode can also be inserted into the brain tissue of rats to detect O_2_, and their neuronal activities can be studied through measured changes in O_2_ concentration [39]. The sensor in [40] demonstrated a response time of approximately 30 s and detected both NO and NO_2_ in vivo, achieving a detection limit for NO_2_ of as little as 5 ppm. The utility of biodegradable materials in gas detection applications also presents significant benefits, with promising implications for use in medical diagnostics and treatment strategies.

Among the studies of gas measurement using probes, in addition to electrochemical sensor probes, the use of fluorescent probes represents another important method for the study of gases in biological tissues because of the high selectivity, nondestructive nature, and excellent biocompatibility of these probes. The fluorescence intensity of the fluorescence probe is proportional to the concentration of the detected object, which can be used to measure the target object quantitatively. Fluorescent probes that are excited and emit in the near-infrared region offer advantages of low light damage and strong tissue penetration, and they thus play an important role in the detection of gas in biological tissues. Wu and colleagues introduced a biocompatible fluorescent probe that used a bovine serum albumin scaffold. This probe enabled effective fluorescence imaging of ethylene within carnation petal tissues and determined the ethylene concentration in these tissues accurately [41]. An experiment conducted by Liang et al. applied a near-infrared fluorescent probe composed of CHO-OH-NO_2_ to enable the rapid and convenient detection of H_2_S in budding wheat seeds. H_2_S plays an important role in plant resistance to Al^3+^-induced stress. In their experiments, the probes were used to monitor the stress levels induced by different concentrations of aluminum ions during the early stages of wheat seed germination, and changes in the amount of H_2_S produced were observed directly [42]. Chen’s team developed three types of fluorescence-labeled Rh (III)-based probes using CEP-1 for the selective and quantitative detection of ethylene to allow them to detect changes in ethylene synthesis in plant cells, with a detection limit of as little as 52 ppb. The experiments proved that this probe offers obvious advantages in terms of detection limit, response time, and selectivity [43]. In addition to H_2_S and NO, CO can also be detected in living cells using corresponding fluorescent probes. As a gas signaling molecule in biological systems, studying the concentration changes in CO is highly significant for biological activities [44]. However, the fluorescence probe also has shortcomings that include a short lifetime and a limited distribution area, and improving the performance of related gas sensors requires further study.

In addition to these measurement applications in biological tissues, porous structures that contain gases with research and measurement significance generally have a porous organic framework; these materials usually have high gas adsorption properties, a large specific surface area, high porosity, and stable chemical properties, and their gas adsorption concentrations can be analyzed using a variety of methods. As one example of a type of porous nanomaterial, metal–organic skeleton material (MOF) has been used as a VOC pre-enrichment material and can also be used as an SPME material to pre-extract volatile organic gases from soil [45,46]. MOFs are pivotal in gas sensor applications because of their composition, which combines metal clusters and organic linkers. Furthermore, nanocomposites synthesized from ZnO/CuO that are derived from MOFs can act as sensors for H_2_S resistance measurement. Their gas adsorption properties allow the resistance value to indicate both the presence and the concentration of the adsorbed H_2_S [47]. Covalent organic frameworks (COFs), which are also a type of porous organic framework, have similar gas adsorption properties and can be used as gas adsorption materials. In [48], Zhang et al. studied the adsorption gas detection performance in a gap, explored the use of the probe detection method to analyze adsorption gas intensity, and also proposed the use of nano spin sensors to perform the analysis. Gaseous adsorption in a pyrene-based COF (Py-COF) was detected using metallic fullerenes with high chemical stability and embedded electron-spun Sc_3_C_2_@C_80_ as the probe. The gas adsorption performance of Py-COF, with respect to different gases (including N_2_, CO, CO_2_, CH_4_, C_3_H_6_, and C_3_H_8_), is reflected by changes in electron paramagnetic resonance signal intensity. With the development of these porous organic materials, the porous materials themselves can be used as gas sensors to reflect the adsorbed gas concentration.

The invasive nature of the detection of gas using probes means that, to some extent, there will be an effect on the properties of the sample; for example, the plant will produce a gas change in response to the damage caused when the electrochemical sensor electrode is implanted. Consequently, when using these probes for gas detection, it is necessary to consider whether the gas within the sample gap will change because of the intrusion of the probe during the measurement, and whether this change will affect the measurement results.

### 3.2. Biosensors

Biosensors are mainly composed of biomolecules such as proteins and peptides that can capture the corresponding gas, and the signal generated by the resulting combination of the gas with the biomolecules of the biosensor is then transmitted to the system for the analysis of the concentration of the target gas. As a gas detection method, gas biosensors play an important role in the detection of specific gases, and the selectivity of this sensor type is an important advantage when using biosensors for gas detection [49]. Vong’s research team developed an ethylene-sensitive enzyme biosensor probe to detect the ethylene levels in various fruits and Arabidopsis plant tissues and achieved a detection limit of approximately 27 ppm [50]. However, their probe’s slow reaction rate may result in the volatilization of ethylene from the plant tissue into the atmosphere during the measurement, leading to an inadequate real-time measurement performance. In contrast, Capo’s team created a biosensor chip that integrated odor-binding proteins to enable the real-time monitoring of benzene with a detection limit of 5 µg/m^3^ [51]. In terms of biosensor detection selectivity, because of the high research significance of NO as a signal molecule for the physiological processes in plants, Wei Wen’s team fixed hemoglobin, chitosan, graphene, and a cetyltrimethylammonium bromide surfactant on a sensor electrode and developed a NO biosensor for NO measurement in plants. Their NO biosensor can still achieve a highly sensitive detection of NO at levels as low as approximately 0.50 μM when interfering substances such as H_2_O_2_ are present in the plant sample [52]. In the field of biomedicine, biogas sensing technology can monitor various gases inside the human body, such as carbon dioxide, oxygen, ammonia, carbon monoxide, etc. The concentration of these gases can be used to reflect the metabolic status of the human body, providing important information for diagnosis, treatment, and monitoring. For example, for the detection of the interstitial gases in biological tissues, biosensors generally offer the advantages of good biocompatibility and reduced impact when inserted as probes into tissues. However, although the selectivity of biosensors allows some gases to be identified with high sensitivity, there are some limitations in the use of biosensors for the detection of complex gases.

## 4. Measurement Method and Technology of Scattering Medium Absorption Spectroscopy

Methods based on absorption spectroscopy, such as the photoacoustic spectroscopy technique, have obvious advantages for use in nondestructive testing because scattering does not cause signal losses and the method is not affected by background light interference [53], and relevant information about a variety of gases can sometimes be determined simultaneously [54]. In the realm of gas detection within porous gas-bearing media, photoacoustic spectroscopy has been used successfully to measure CO_2_ in tree rings [55] and to measure CH_4_ and N_2_O gases in soil pores [56]. Infrared spectroscopy, when used in conjunction with photoacoustic gas instruments, can identify trace gas molecules at concentrations within the range from 1 to 100 ppm to perform both qualitative and quantitative evaluations [57,58]. The detection limit of the photoacoustic spectrum for trace gases ranges up to the ppb level, thus enabling the sensitive detection of gas concentration, but the method has the disadvantages of low precision, high cost, and short lifetimes for complex gas measurements [59]. Fourier-transform infrared (FTIR) spectroscopy offers high sensitivity [60] down to the ppb level and provides a real-time analysis capability that is suitable for field applications. However, errors arise when FTIR is used in quantitative assessments to deal with complex compounds. In addition, tunable diode laser absorption spectroscopy (TDLAS) is an absorption spectroscopy technique that has been used to monitor trace gases. The advantage of this method is that it can vary the laser wavelength, which allows the detection of all substances absorbed within the infrared region, and it also has a low detection limit that can reach the ppb level. Although TDLAS also has certain limitations for the detection of complex compounds, it has been applied in the monitoring and analysis of diffused alkane gas caused by oil leakages in soil gaps [61].

For the analysis of solid media with gas-filled pores, one absorption spectroscopy method uses the distinctive characteristics of the medium; specifically, the spectral features of the gas within the pores are more pronounced than the corresponding features of the medium itself. By analyzing absorption spectrum characteristics, it is possible to deduce the gas composition that is present in the pore spaces. This technique, which is known as gas scattering medium absorption spectroscopy (GASMAS), was initially proposed by Svanberg’s research team at Lund University in Sweden and has been explored for use in various applications. For example, GASMAS is capable of measuring gases trapped within porous scattering materials, including wood [62], fruit, and foam, and the process also holds significant potential for the assessment of gaseous content in biological tissues.

**Figure 4:**
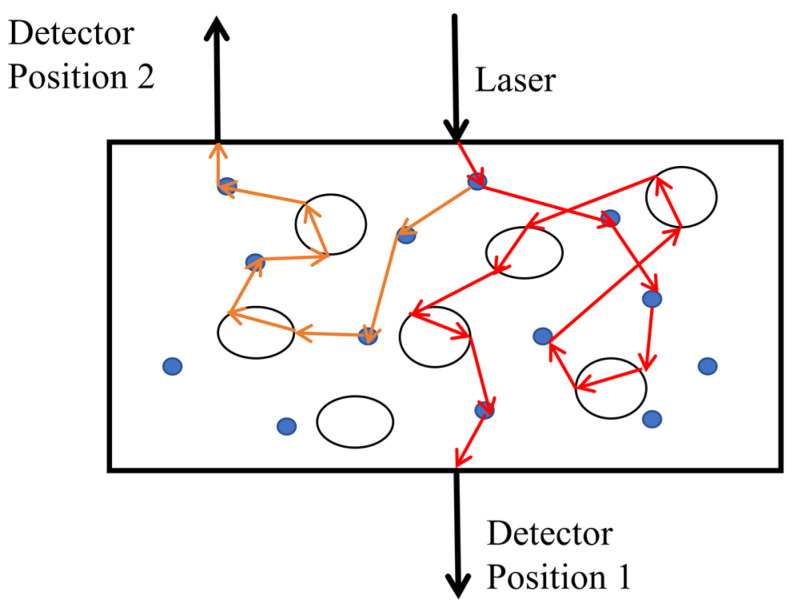
Optical path simulation and detector positioning in scattered material.

During measurement experiments, the laser emission wavelength encompasses the absorption wavelength range of the target gas. The detector can be positioned on the same side of the sample as the laser to capture scattered light reflection signals, or it can be placed on the opposite side of the sample, facing the laser, to receive the transmitted signals. The simulated optical path within the scattering medium and the associated receiver positioning are illustrated in Figure 4. The scattered light can be detected at either detector position 1 or position 2, thus providing a degree of flexibility for the adjustment of both the laser and receiver locations based on the experimental conditions. For example, when monitoring the gas concentrations in an egg [63] and a femoral head [64], comparison measurements were conducted by varying both the laser injection and light-receiving positions, demonstrating that the absorbed signal strengths remained largely consistent across these configurations. This indicates that relative positioning has a minimal impact on measurement outcomes. However, it is essential to ensure that the received light avoids interference from the strong emissions that occur at the laser emitter’s injection point; therefore, it is essential to maximize scattering to minimize signal disruption.

Given that the scattered light signals are inherently weak, the accurate extraction of gas information requires wavelength modulation of the laser—by transferring the signals onto a high-frequency carrier frequency—to mitigate the influence of noise. Subsequently, phase-locked amplification technology is used to perform demodulation and yield second harmonic signals (2*f*) for use in data processing.

One of the main problems with use of the GASMAS technique to analyze gases in solid pores is that the path of the laser light through the gas in the scattering medium is uncertain; there may be several different scattering paths at the same time, and the path length is difficult to measure directly, which means that the gas concentration cannot be obtained directly using the Beer–Lambert law. Therefore, to determine the concentration of a gas, the absorption of light through this gas over a distance is usually used for reference; this optical path is recorded as the average equivalent path length ($l_{eq}$), which corresponds to the path length of the light when propagating in a gas of known concentration (e.g., in an air environment), and the same absorption is produced when propagating in a porous medium. For example, when $c_{airO_{2}}$ is the oxygen concentration in the air, $c_{sample}$ is the oxygen concentration in the sample, and $l_{sampleO_{2}}$ represents the actual optical path length through the sample:

(1) $$l_{eq}\times c_{air\;O_{2}}=l_{sample}\times c_{sample\;O_{2}}$$

The absorption of light when it passes through oxygen for a distance of $l_{sample\;O_{2}}$ with a concentration of $c_{sample}$ in the sample is equivalent to the absorption of light when passing through oxygen for a distance of $l_{eq}$ in air. In the experimental measurements, the gas absorption measured by the GASMAS system is expressed as $l_{eq}$, which can also be expressed as the product of relative gas concentration (%) and absorption path length using Equation (2):

(2) $$l_{eq}=l_{sample}\frac{c_{sample\;O_{2}}}{c_{air\;O_{2}}}$$

**Figure 5:**
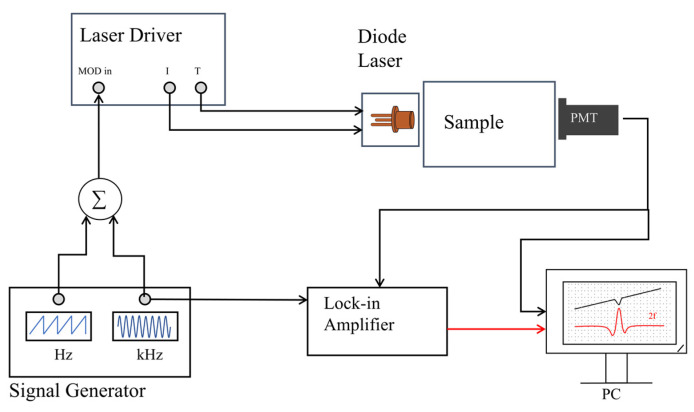
Schematic diagram of the connections in the GASMAS experimental setup.

A device diagram of the GASMAS system is shown in Figure 5. The signal generator generates both a low-frequency sawtooth signal and a high-frequency sinusoidal signal. The low-frequency signal is used to scan the absorption wavelength of the gas to be measured to obtain the absorption signal and the high-frequency sinusoidal signal is used to perform the wavelength modulation. The two signals are superimposed to act as the input signal for the laser driver device to adjust the laser’s temperature and drive current. The directly absorbed signal that is received by the photomultiplier and the 2*f* signal that is demodulated by the phase-locked amplifier are both transmitted to the computer for recording and subsequent data analysis.

There are several methods that are commonly used in experiments to determine the gas concentration in a sample, including the following examples.

The first method involves the selection of a gas with a known concentration, e.g., water vapor, to act as a reference gas when measuring the unknown concentration of the gas in the sample. At this time, the laser must scan the water vapor and the absorption wavelength of the gas to be measured, i.e., two lasers can be selected to measure the sample simultaneously. When the sample to be tested is saturated with water vapor, the water vapor concentration can then be calculated based on the ambient temperature parameters using the Arden Buck relationship. At this time, it is assumed that the volume of gas within the pores of the medium is the same, i.e., the optical path of the laser beam through the water vapor is regarded as being the same as the optical path through the gas to be measured, and the oxygen concentration is then calculated according to the path length obtained. It should be noted that this assumption can only be made if the wavelengths used to interrogate the two gases are very similar [65]. At this time, according to Equation (1),

(3) $$\frac{l_{eq\;gas\;}}{l_{eq\;H_{2}O\;}}\frac{c_{ref\;gas\;}}{c_{ref\;H_{2}O\;}}=\frac{l_{\;gas\;}}{l_{\;H_{2}O\;}}\frac{c_{\;gas\;}}{c_{\;H_{2}O\;}}$$

Among these parameters, $l_{gas}$ and $l_{H_{2}O}$ are regarded as being as the same, $c_{H_{2}O}$ can be determined based on the temperature, and $l_{eqgas}$ and $l_{{eqH}_{2}O}$ are the absorbed signals measured by GASMAS system. $c_{refgas}$ and $c_{{refH}_{2}O}$ represent the concentrations of the gas and the water vapor in the reference environment, respectively, and can be measured or expressed as relative gas concentrations (%). Therefore, the concentration $c_{gas}$ of the gas to be measured can be expressed.

However, when using water vapor as the reference gas to calculate the concentration of the target gas, it is essential to note that the direct calculation of the water vapor concentration using the Arden Buck relation requires the water vapor to be saturated in the prevailing environment. Consequently, this method is typically used in scenarios in which liquid water is present within the sample to ensure water vapor saturation in the sample gap. For example, in cases that involve sensing gases emitted from baked bread sealed within packaging, the use of water vapor as the reference gas is inappropriate because of the non-saturated state [66]. Furthermore, during oxygen concentration measurements within ground meat gaps, it should be noted that when the temperature increases gradually during the measurement process, the saturated water vapor concentration varies with the temperature; therefore, the measured water vapor $l_{eq}$ shows an obvious signal enhancement, but it does not reflect the real change in the oxygen concentration. It is thus necessary to normalize the water vapor signal to exclude the effects of temperature. Therefore, when measuring the gas in the sample, the temperature conditions should remain as stable as possible.

This approach—involving treating water vapor as a reference gas—is generally applicable to the analysis of samples saturated with moisture content, such as the interstitial gases found in human tissues, including those within the lungs and the femoral head pores. Since the inception of the GASMAS technique, researchers have actively pursued the development of its medical applications. In 2014, Pacheco et al. established an initial model that simulated the optical characteristics of the human chest and lung structures with the aim of exploring potential detection capabilities using GASMAS technology for tissue gas concentrations [65]. By 2021, his team had refined this model by incorporating more precise optical properties specific to alveoli and conducted experiments under simulated physiological conditions to demonstrate the feasibility of using GASMAS to measure oxygen levels in newborn lungs [67]. Additionally, in reference [68], piglet lung models were developed alongside induced monitoring experiments that covered various lung disease states to confirm the applicability of GASMAS in tracking pulmonary oxygen levels amidst various pathological conditions. Moreover, 2022 saw further investigations into neonatal lung status via monitoring experiments using this non-invasive technique, which offers distinct advantages over alternative methods [69]. Moreover, the explorations of Svanberg et al. into the extension of GASMAS applications to adult pulmonary assessments revealed certain limitations that warranted additional research into device enhancements to allow the technique to be tailored specifically for adult monitoring purposes [70]. Given that bone tissue comprises numerous small gaseous cavities, the use of GASMAS systems consequently allows the examination and analysis of the internal pore gases located within femoral heads—a medically valuable endeavor that aids in the evaluation of patient femoral health status [64]. Chen et al. focused on the assessment of variations pertaining to the humidity signals from the surrounding space around necrotic femoral heads, which indicated significantly diminished readings when compared with osteoarthritis-free patients and highlighted discrepancies related to the gaseous compositions observed therein; however, some specimens exhibited elevated humidity signals that were indicative of underlying pathologies, thus providing critical insights that were relevant to the diagnosis of femoral head diseases [71].

In addition to the treatment of water vapor as a reference gas, the gas of interest itself can also be measured directly to act as a reference. The second most commonly used measurement method is measuring the gas concentration before and after a specific period of time, and then using the gas concentration in the case of equilibrium with the gas concentration in the environment, as recorded before performing the measurement, as a reference [72]. At this time, the gas concentration can then be calculated according to Formula (4). For example, to measure the oxygen concentration between pears, the method uses the following equation:

(4) $$\frac{l_{eq\;0\;}}{l_{eq\;1\;}}\frac{c_{ref\;gas\;}}{c_{ref\;gas\;}}=\frac{l_{\;0\;}}{l_{\;1\;}}\frac{c_{\;0\;}}{c_{\;1\;}}$$

where $c_{0}$ denotes the O_2_ level of the initial sampled gap and $c_{1}$ reflects the subsequent time-point reading, $l_{0}$ is equal to $l_{1}$, and the fixed parameter $c_{ref\;gas}$ represents atmospheric O_2_ composition. This yields the resultant finding:

(5) $$c_{1}=c_{0}\times \frac{l_{\;eq\;1\;}}{l_{\;eq\;0\;}}$$

GASMAS is used to study the interstitial gases in fruits, and studies of the concentration changes in these gases produced by respiration in fruits are conducive to providing guidance for preservation via air conditioning during transportation. Before studying the intercellular gas concentrations of pears, Joseph et al. established an optical model of a pear [73], simulated the scattering coefficients of the different tissue layers (the peel, pulp, and core), and then simulated the resulting light scattering situation. The GASMAS system was subsequently used to measure the change in oxygen concentration between peeled and unpeeled apples, and the effect of the peel on oxygen diffusion behavior was studied [74]. Joseph et al. also measured the interstitial oxygen diffusion behavior of apples when coated with a preservation layer and proved that the gas diffusion rate of the apples coated with this preservation coating was significantly lower than that of uncoated or peeled apples, thus confirming the effect of the preservation layer coating in extending the storage time of the fruit [75].

In terms of research on the interstitial gases produced during the ripening processes of various fruits, previous studies selected four different fruits (nectarines, mangos, papayas, and guavas) as measurement objects to study their specific respiratory characteristics. GASMAS technology was then used to record the gas concentrations in the interstitial pulp space during the ripening process, from immature to ripe. According to the change law of the oxygen concentration during the ripening process, the concentration first decreased before increasing slightly. The point at which the oxygen concentration began to rise slightly was then selected as the threshold marker for full ripening, the maturity of the fruits was evaluated according to the observed law [76], and the spoilage of the different fruits was then predicted based on this law [72].

The advantages of using the GASMAS principle to measure target gases are that gas concentrations can be detected without damaging or destroying the sample and gas concentration changes can also be monitored and recorded in real time. However, it should be noted that the difficulty with using the GASMAS system for the processing and analysis of laser signal information is that the laser source signal received by the detector is very weak because of the complex scattering path of the light in the sample; therefore, it is necessary to use wavelength modulation technology to process the received signal before analyzing it. It is generally necessary to normalize the signal modulated by the wavelength modulation technique, i.e., the amplitude of the 2*f* signal is divided by the light intensity received by the detector, which is used as the value of $l_{eq}$. In addition, to minimize noise interference, mechanical vibration is usually introduced into the transmission device or the sample to eliminate the effects of interference fringes caused by reflection between the surfaces of the optical components. One method that is often used when studying changes in gas concentration during gas exchange inside a sample involves initially placing the sample in a nitrogen environment for a suitably long time, and then moving it to an air environment to observe the changes in the gas concentration in the gap.

In addition, a distant version of GASMAS was introduced in [77], in which LIDAR-GASMAS was used to measure the gas in snow gaps and to monitor the gas leakage that may have been hidden under snow. In addition, the GASMAS proof-of-principle experiment used a porous material such as alumina ceramic to act as a gas chamber and was able to measure the concentration of methane gas that filled this chamber. When the average time was around 20 s, the detection limit could reach 4.5 ppm [78]. In summary, the non-invasive nature of the GASMAS technique represents a significant advantage for the detection of gas concentrations in dispersed porous media, and thus offers valuable potential for applications in areas including gas sensor technology, biomedical fields, and food packaging. Currently, the GASMAS measurement system is predominantly used for the assessment of water vapor and oxygen, thus indicating the need for further advancements to enable its use in the measurement of various other gases.

## 5. Conclusions

In this paper, the methods for the detection of gases that are present in porous media are summarized, the detection methods of related instruments that can perform rapid nondestructive testing in the field are summarized, various sensing technologies are compared and evaluated, and their measurement characteristics and application scenarios are analyzed. In recent years, field analysis instruments have seen rapid development, and associated gas sample collection and pre-treatment methods have promoted the development of field detection. Combining these different detection instruments and methods has improved the accuracy of gas analyses by retaining their respective advantages. As a result of ongoing developments in materials science, the various types of measurement instruments and sensors are tending to be miniaturized, and are thus more suitable for use in practical application scenarios. Invasive measurement methods play an important role in the study of biological activities, and real-time monitoring is highly significant when measuring the states of biological activities.

With recent developments in sensing technology, headspace dynamic acquisition and intrusive acquisition methods are becoming increasingly diversified. Their sampling methods, which take the effectiveness and convenience of the sampling process into account, are of great interest. In terms of pre-treatment methods, pre-concentration methods are now more abundant, allowing faster pre-concentration processes to be realized, greatly improving the detection limits of portable instruments and promoting the development of field-testing instruments. In terms of detection methods, non-invasive gas detection methods are becoming increasingly accurate. The development of porous materials, which act as sensors to react directly to the concentration of the attached gas, and other characteristics has provided new ideas for process enrichment and for performing measurements within measurements, and has also resulted in the development of gas sensors. In view of the difficulties involved in performing gas sampling or measurements in porous media, in addition to the assessment of direct or indirect gas detection approaches, modeling methods have also begun to be used to analyze gas concentrations within large areas, and are no longer limited to sampling and detection applications. In terms of the environmental changes caused by the instability of sensors and their wide application, the development of gas sensing technology is still facing certain technical challenges, but with the exploration of gas sensing in materials science, combined with new nanomaterials and metal–organic framework compounds, gas sensors are developing in the direction of miniaturization, integration, and intelligence. The development requirements for improving accuracy can be gradually realized. In short, in terms of the methods used to acquire gas information in porous media, we have highlighted the specific advantages and limitations experienced in the generation and development of each measurement method. When the measurement object is varied, the most appropriate measurement method can be used to meet the measurement demands. The methods available to acquire the gas components contained in porous media are becoming increasingly diverse, with their development leading toward fast detection speeds and high measurement precision. The monitoring of gas in porous media in the natural environment is very important for the timely detection and pollution source control of explosion and poisoning accidents caused by harmful gases and soil pollution caused by leakages. This is of great significance to the realization of resource safety and personnel and equipment safety. The monitoring of gases in food and crops and the accurate control of their quality are conducive to the improvement of production and reductions in costs, and play an important role in the economic growth of agricultural products. In biomedicine, the monitoring of gas in living organisms is of great significance for the treatment and diagnosis of diseases, as well as the assessment of life activities and characteristics. In summary, gas sensors play an important role in many fields, such as environmental monitoring and industrial safety, food safety, and agricultural cultivation, and are of great significance in achieving environmental safety assurance and the economic growth of agricultural products.

## Data Availability

The authors confirm that the data supporting the findings of this study are available from the corresponding author upon reasonable request.
